# Multivariate classification of urine metabolome profiles for breast cancer diagnosis

**DOI:** 10.1186/1471-2105-11-S2-S4

**Published:** 2010-04-16

**Authors:** Younghoon Kim, Imhoi Koo, Byung Hwa Jung, Bong Chul Chung, Doheon Lee

**Affiliations:** 1 Department of Bio and Brain Engineering, KAIST, Daejeon, South Korea; 2 Korea Institute of Oriental Medicine, Daejeon, South Korea; 3 Bioanalysis and Biotransformation Research Center, KIST, Chengryang, Seoul, South Korea

## Abstract

**Background:**

Diagnosis techniques using urine are non-invasive, inexpensive, and easy to perform in clinical settings. The metabolites in urine, as the end products of cellular processes, are closely linked to phenotypes. Therefore, urine metabolome is very useful in marker discoveries and clinical applications. However, only univariate methods have been used in classification studies using urine metabolome. Since multiple genes or proteins would be involved in developments of complex diseases such as breast cancer, multiple compounds including metabolites would be related with the complex diseases, and multivariate methods would be needed to identify those multiple metabolite markers. Moreover, because combinatorial effects among the markers can seriously affect disease developments and there also exist individual differences in genetic makeup or heterogeneity in cancer progressions, single marker is not enough to identify cancers.

**Results:**

We proposed classification models using multivariate classification techniques and developed an analysis procedure for classification studies using metabolome data. Through this strategy, we identified five potential urinary biomarkers for breast cancer with high accuracy, among which the four biomarker candidates were not identifiable by only univariate methods. We also proposed potential diagnosis rules to help in clinical decision making. Besides, we showed that combinatorial effects among multiple biomarkers can enhance discriminative power for breast cancer.

**Conclusions:**

In this study, we successfully showed that multivariate classifications are needed to precisely diagnose breast cancer. After further validation with independent cohorts and experimental confirmation, these marker candidates will likely lead to clinically applicable assays for earlier diagnoses of breast cancer.

## Background

Breast cancer is currently the second most common type of cancer [1] after lung cancer and the fifth most common cause of cancer death [2]. Therefore, with the appearance of many high-throughput measurement technologies, there have been many studies of the diagnosis of breast cancer using high-throughput methods of analysis. Samples for the diagnostic analysis of the breast cancer include urine, serum, plasma, or tissue, and various components are measured, including mRNA, proteome, metabolome, epigenome. 

Of the various types of samples, diagnostic techniques using urine are advantageous in terms of clinical application to real patients because these techniques are non-invasive, inexpensive, and easy to perform, likely leading to earlier detection for malignancies [3]. In addition, since metabolites are end products of cellular processes, their concentrations reflect the systems-level response of biological systems and are closely linked to phenotypes and diseases [4]. Urine, moreover, contains many classes of compounds, including organic acids, amino acids, purines, pyrimidines, sugars, sugar alcohols, sugar acids, and amines, which can be diagnostic clues for a variety of abnormalities. Therefore, urine metabolome is very useful in biomarker discoveries and clinical applications. However, only univariate methods such as a t-test, chi-square, and ANOVA have been used in classification studies using urine metabolome [5-11]. Principal Component Analysis (PCA) or Partial Least Squares (PLS) methods, which is a multivariate method, also has been used, but it is, as a dimension reduction method, not meant for constructing classification models, but for visualizing overall distributions of given data or examining separability between different groups.

**Figure 1:**
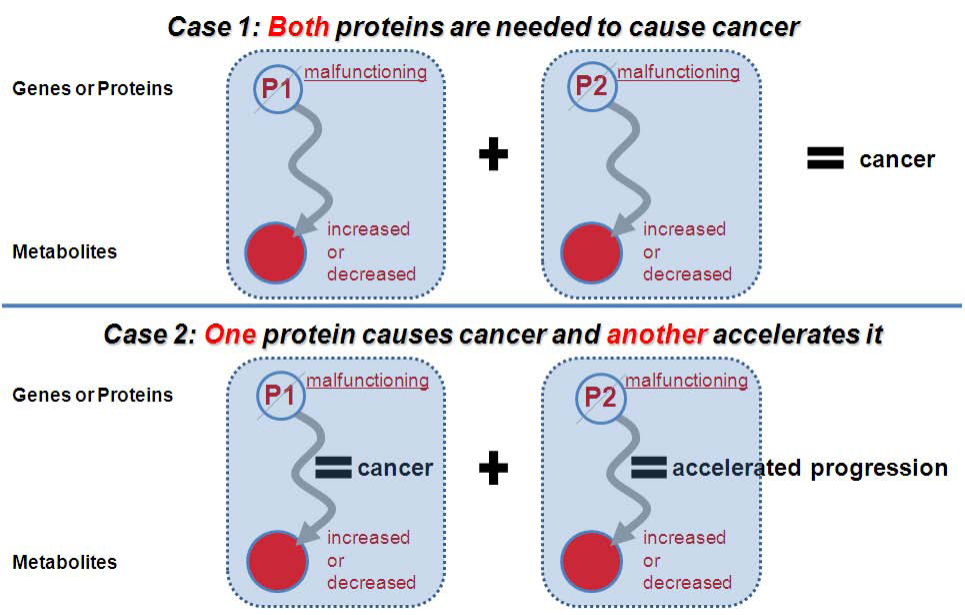
**Potential cases in which multiple proteins are simultaneously related to cancer developments** In the case one, two metabolites should be measured simultaneously to identify cancer. Both metabolites also should be detected in the case two for accurate diagnosis. Therefore, these cases show that only one metabolite may not be enough for cancer diagnosis.

Since multiple genes or proteins would be involved in developments of complex diseases such as breast cancer, multiple compounds including metabolites would be related with the complex diseases, and multivariate methods would be needed to identify those multiple metabolite markers. Moreover, because combinatorial effects among the markers can seriously affect disease developments and there also exist individual differences in genetic makeup or heterogeneity in cancer progressions, single marker is not enough to identify cancers. Figure 1 shows multiple components involved in cancers and combinatorial effects among them. However, there have been no multivariate classification studies for urine metabolome data. Although Denkert et al. [12] performed multivariate-based classifications for metabolome data, they used tissue metabolome datasets. Besides, they did not consider biological implications of multivariate classifications in the paper.

**Figure 2:**
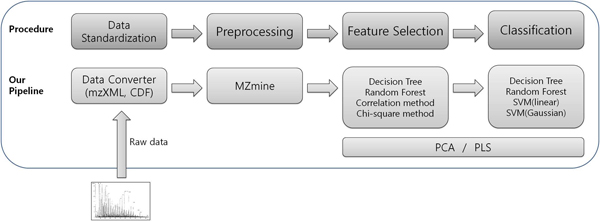
**An overview of the analysis procedure used to construct classification models based on metabolome datasets** The procedure consists of four stages; data standardization, preprocessing, feature selection, and classification. The raw data from mass spectrometry machines are converted into the standard data formats mzXML [13] and CDF, and in turn preprocessed using the MZmine tool [14,15]. The data are then analyzed with various feature selection and classification techniques. For feature selection, we use chi-square as a univariate method, the correlation-based method as a multivariate method, and Decision tree and Random forest as classifier-embedded methods. For classification, we use Decision tree and Random forest as tree-based non-parametric methods and Support vector machine (SVM) as a generalized linear discriminative method. (An Artificial neural network (ANN) is not used here, since it is known that the ANN has weak points in many cases, compared to the SVM [18,19].) The dimension reduction methods PCA and PLS are used for visualizing overall distributions of given data.

**Figure 3:**
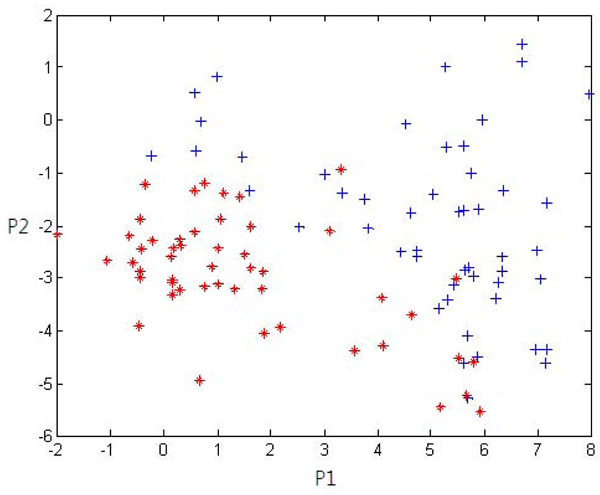
**A result of partial least square (PLS) for the given data** The datasets are gas chromatography coupled to mass spectrometry (GC-MS) profiles from urine samples of 50 breast cancer patients and 50 normal women. The crosses represent cancer patients, while the asterisks represents normal women. Two classes are separated well on two principal component axes. This result suggests a high likelihood that urine samples have separability between normal women and breast cancer patients.

**Figure 4:**
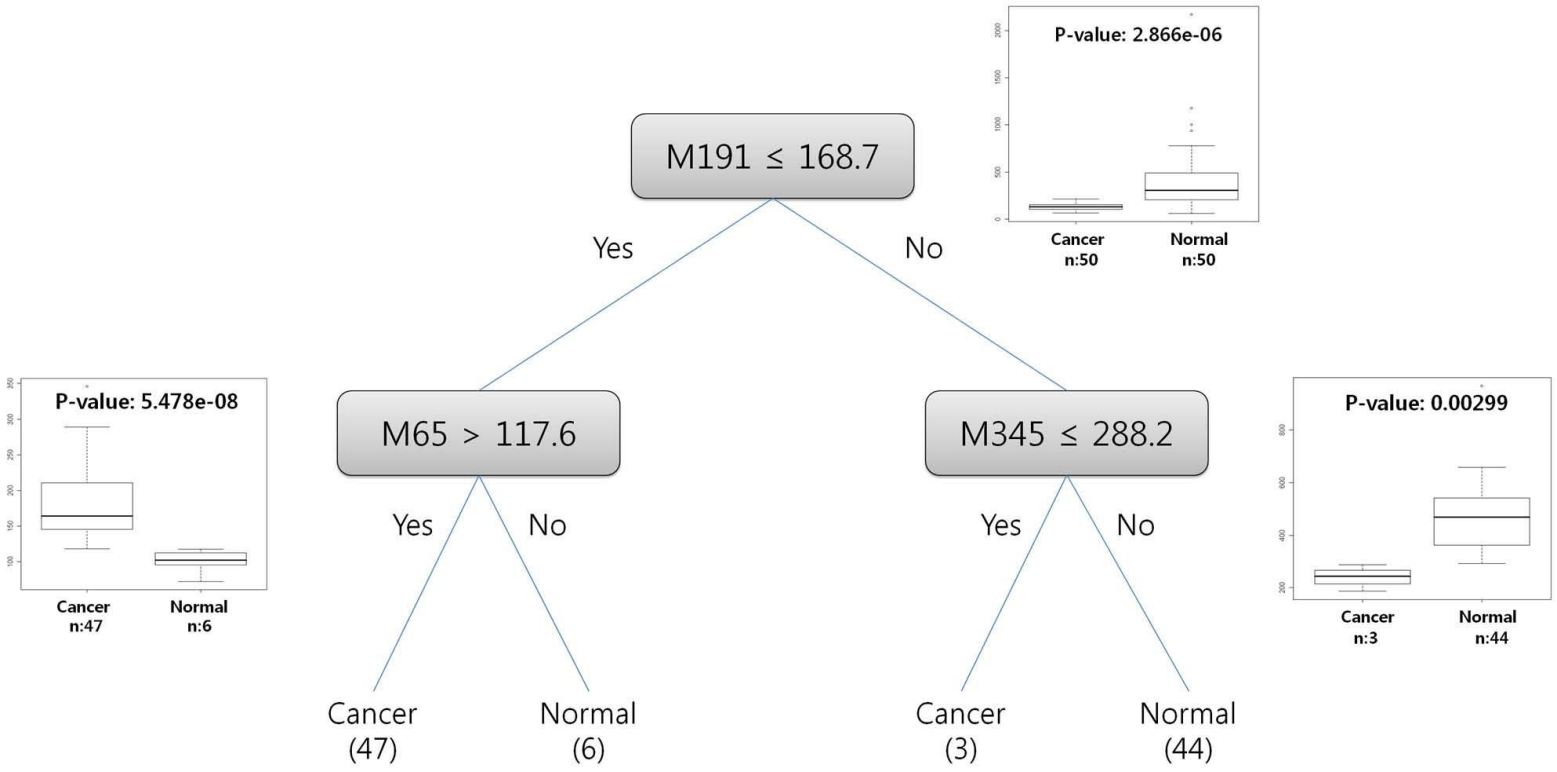
**The proposed classification model using the first dataset (Table **1A**)** This model was constructed by Decision tree. The dataset consists of three features: M191, M65, and M345. The rectangles represent nodes in a tree, and the box-plots are the corresponding t-test results of each node, including their p-values, showing the discriminating power of the features.

**Figure 5:**
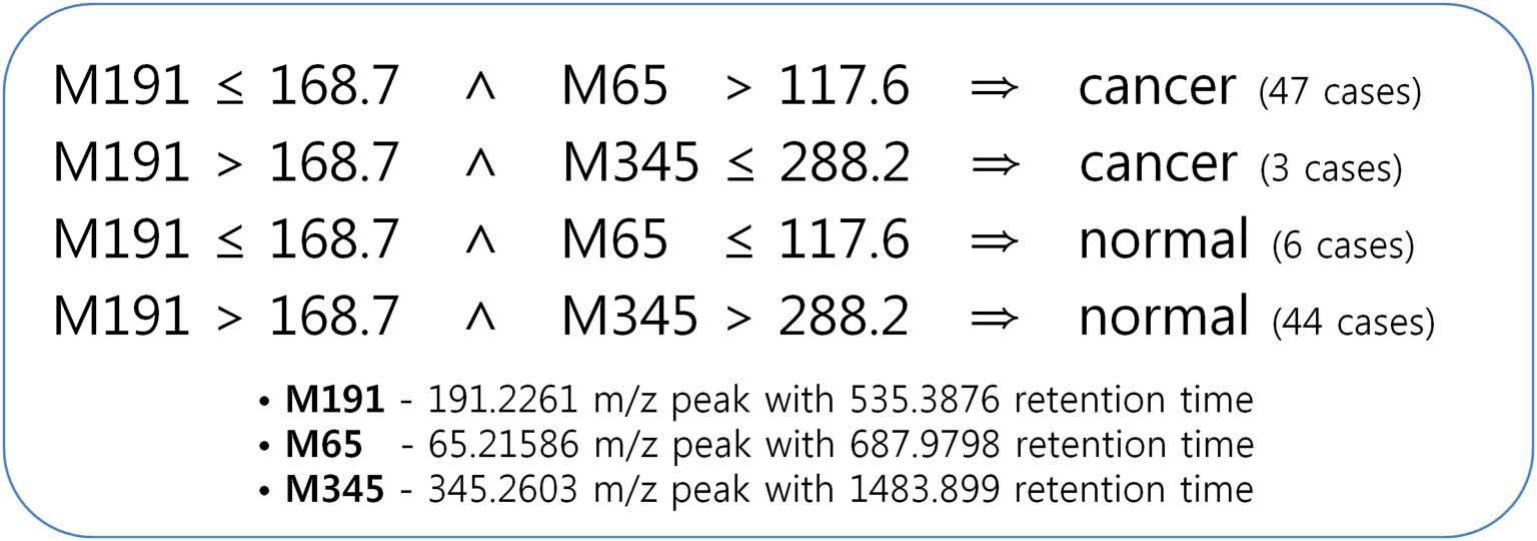
**Potential diagnosis rules to help clinical decision making for breast cancer** These rules are derived from the classification model by Decision tree for the first dataset (Table 1A). The values indicate intensities of mass spectrometry of each peak.

**Figure 6:**
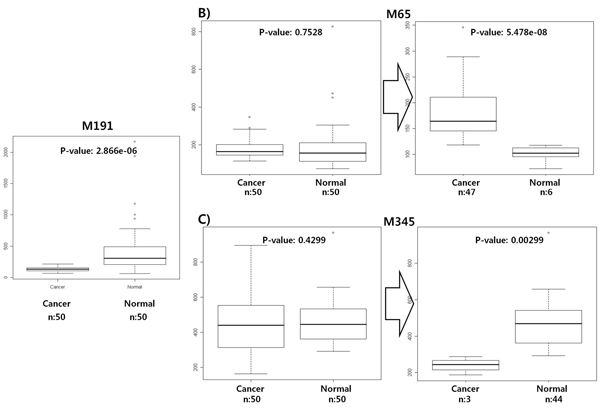
**The comparison of the performance of univariate versus multivariate classification** A t-test, which is a univariate method, has been applied to the identified feature sets. A) M191 shows a p-value of 2.866e-06 in the t-test, and it seems that this peak can be identified by both of the univariate and multivariate method. B) However, M65 shows a p-value of 0.7528; therefore this peak cannot be identified by only the univariate method. In this case, if the multivariate method is applied, then M65 can be identified, because the multivariate method considers more than two features at the same time and, that is, can find the case that breast cancer and normal samples are classified if two conditions should be satisfied simultaneously; here, the intensity of M65 is more than 117.6 and the intensity of M191 is less than 168.7. It seems that this discriminative power of multivariate methods is highly appropriate for biological systems in which more than dozens of factors are able to affect single disease. As a result, since both conditions are applied together, the criterion becomes strict and the p-value of M65 is dramatically decreased from 0.7528 (left boxplot; by univariate method) to 5.478e-08 (right boxplot; by multivariate method). C) The p-value for M345 has also been decreased from 0.4299 (left) to 0.00299 (right).

**Figure 7:**
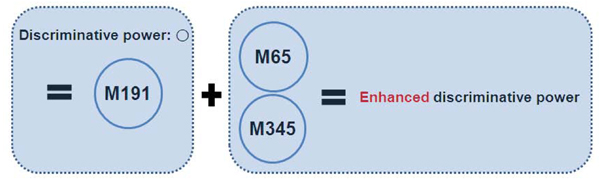
**M191 causes cancer and M65 and M345 accelerate it** In the results, M191 itself had enough discriminative power for breast cancer, but when it worked together with M65 and M345, the discriminative power was considerably enhanced. This is the second case of the figure 1 and shows that there exists an issue of significant combinatorial effects among multiple metabolites in real dataset analysis.

Therefore, in this study, we proposed classification models using multivariate classification techniques (Figure 2) and developed an analysis procedure for classification studies using metabolome data. (Figure 3) Through this strategy, we identified five potential urinary biomarkers for breast cancer with high accuracy, among which the four biomarker candidates were not identifiable by only univariate methods. (Figure 4, Table 1, 2, 3) We also proposed potential diagnosis rules to help in clinical decision making. (Figure 5) Besides, we showed that combinatorial effects among multiple biomarkers can enhance discriminative power for breast cancer. (Figure 6 and 7)

**Table 1:** A list of selected feature sets in the feature selection stage

The best feature set (A)				The second feature set (B)				Univariate-based feature set(C)		
Depth of nodes in Decision Tree	m/z	RetentionTime(sec)	Rank	Depth of nodes in Decision Tree	m/z	RetentionTime(sec)	Rank	m/z	RetentionTime(sec)	Rank

0	191.2261	535.3876	1	0	191.2261	535.3876	1	191.2261	535.3876	1

1	65.21586	687.9798	4138	1	93.22983	551.3150	2839	401.1959	781.4042	2

1	345.2603	1483.899	5229	2	147.2395	277.6261	1074	311.2211	783.1188	3

The best feature set (A), the second-best set (B) by the multivariate feature selection method, and a feature set (C) by the univariate method for comparison. Rank in the feature list from the univariate feature selection is shown.

**Table 2:** Contingency table for the first feature set

	Actual	Output		Accuracy	Sensitivity	Specificity
					
		Cancer	Normal			
**Decision Tree**						
Confidence=0.25	Cancer	47	3	94.00%	94.00%	94.00%
Pruning=true	Normal	3	47			
							
**Random Forest**						
Tree=500	Cancer	47	3	95.02%	94.00%	96.00%
Feature=6	Normal	2	48			
							
**Support Vector Machine (Linear)**				
Cost=1	Cancer	50	0	89.06%	100.00%	72.00%
Gamma=0.33	Normal	14	36	# of Support Vectors: 52	
							
**Support Vector Machine (Gaussian)**				
Cost=45	Cancer	49	1	95.16%	98.00%	92.00%
Gamma=0.33	Normal	4	46	# of Support Vectors: 22	
							
Classification results for the first feature set (Table 1A)					

Contingency table showing number of cases classified for each of the diagnostic classes for the first feature set (Table 1A).

**Table 3:** Contingency table for the second feature set

	Actual	Output		Accuracy	Sensitivity	Specificity
					
		Cancer	Normal			
**Decision Tree**						
Confidence=0.25	Cancer	46	4	90.06%	92.00%	88.00%
Pruning=true	Normal	6	44			
						
**Random Forest**						
Tree=500	Cancer	46	4	91.02%	92.00%	90.00%
Feature=6	Normal	5	45			
						
**Support Vector Machine (Linear)**				
Cost=25	Cancer	48	2	91.41%	96.00%	86.00%
Gamma=0.33	Normal	7	43	# of Support Vectors: 26	
						
**Support Vector Machine (Gaussian)**				
Cost=45	Cancer	46	4	91.02%	92.00%	90.00%
Gamma=0.33	Normal	5	45	# of Support Vectors: 26	
						
Classification results for the second feature set (Table 1B)					

Contingency table showing number of cases classified for each of the diagnostic classes for the second feature set (Table 1B).

## Data

### Urine sample collection

Urine samples were collected from female breast cancer patients (n = 50, age 47.6 ± 7.89 yr) and healthy subjects as normal controls (n = 50, age 46.64 ± 7.38 yr) at the Samsung and Hanyang University Medical Centers (Seoul, Korea). All study subjects underwent the same diagnostic procedures, i.e., a physical examination of the breasts, mammography, and ultrasonography as detailed by the American Joint Committee on Cancer staging. Breast cancer patients underwent either a modified radical mastectomy (MRM) or a lumpectomy with an auxiliary lymph node dissection. Both pre- and postoperative urine samples were collected, with the latter obtained 2 weeks after surgery. The sex- and age-matched controls had no evidence of benign or malignant breast disease. All of the urine samples were collected in the early mornings and kept frozen at −20°C until analysis. In this study, we used only pre-operative and normal samples to construct models to distinguish between breast cancer and normal samples.

### Sample preparation

Urinary metabolites were prepared by extraction under four conditions. First, each urine sample (1 mL) was loaded into a Strata-X cartridge (60 mg, 3 mL; Phenomenox, Torrance, CA), washed with distilled water (1 mL), and extracted with 4 mL of methanol. The eluate was divided in half, and one half of the eluate (2 mL) was evaporated and dried in the desiccator over 30 min. For the second condition, the other half of the eluate was evaporated, dissolved in 1 mL of 0.2 M acetate buffer (pH 5.2), and hydrolyzed with β-glucuronidase/arylsufatase (50 μL) from Helix Pomatia (Roche, Mannheim, Germany) at 55°C for 3 hr. After cooling, urinary metabolites were extracted with 5 mL of diethyl ether by mechanical shaking for 20 min and centrifugation at 2500 rpm for 5 min. The separated organic layer was evaporated under nitrogen and kept in the desiccator over 30 min. For the third condition, the remaining aqueous layer was adjusted to pH 1-2 with 200 μL of 3 M HCl and extracted with 5 mL of diethyl ether. The separated organic extract was evaporated and dried. For the fourth condition, the remaining aqueous layer was adjusted to pH 10 – 11 with 0.73 g of K2CO3 and extracted with 5 mL of diethyl ether and dried. All dried extracts were derivatized by 50 μL of MSTFA/TMSI/TMCS (100:2:5, v/v/v) mixture at 60°C for 15 min and injected into a GC-MS system.

### Instrumental conditions

All samples prepared were separated through a Ultra-1 capillary column (25 m x 0.2 mm ID, 0.33 μm film thickness; Agilent, Palo Alto, CA) and analyzed by a Thermo Finnigan GC-MS system consisting of a Trace 2000 GC and a Polaris Q mass-selective detector in the scan range of m/z 50 – 800 (Thermo Finnigan, Waltham, MA).

## Methods

In this study, we have organized an analysis procedure to construct classification models based on metabolome datasets using various multivariate classification methods. The procedure consists of four stages: data standardization, preprocessing, feature selection, and classification. (Figure 2)

1) Data standardization stage: raw data from the mass spectrometry machine is converted into standard formats. The mzXML [13] and CDF formats are well-known and used in this work.

2) Preprocessing stage: multiple steps are used to preprocess raw data, including smoothing, peak detection, and peak alignment. For those purposes, MZmine software [14,15] is suitable and used in this work; this program is freeware and is appropriate for liquid chromatography coupled to mass spectrometry (LC-MS), gas chromatography coupled to mass spectrometry (GC-MS), and capillary electrophoresis coupled to mass spectrometry (CE-MS).

3) Feature selection stage: it is critical in the construction of classification models and in biomarker discovery to extract the meaningful variables from among thousands of variables (in this work, m/z peaks). To reflect various types of distributions of data, we have used a variety of feature selection algorithms comprising univariate (t-test and chi-square), multivariate (the correlation-based feature selection (CFS) algorithm), and model-embedded methods (Decision tree [16] and Random forest [17]).

4) Classification stage: with selected feature sets, classification models are constructed. In this work, Decision tree and Random forest are used as tree-based non-parametric methods. Support vector machine (SVM) is used as a generalized linear discriminative method. An Artificial neural network (ANN) is not used since it is known that SVM outperforms ANN unless training datasets are sufficient [18], and ANNt is also weak at over-fitting and computational complexity because too many parameters must be estimated [19]. All the algorithms are multivariate.

In addition, for visualization of datasets the dimension reduction algorithms PCA and PLS, are used, allowing separablity of given datasets to be checked.

## Results

### Preprocessing of urine metabolome datasets

We have constructed models to classify urine metabolome data into breast cancer and normal, and we have identified several potential biomarkers for breast cancer, which are detectable in urine samples, with the metabolome-data analysis procedure described above. First, a total of 26,306 features, which are m/z valued-peaks with retention time information, are standardized through our data converter and then preprocessed by MZmine (Smoothing, peak detection, peak alignment, gap-filling, and normalization of the software were performed with default parameters. Peaks with the same m/z value are regarded as different if their retention times are different.).

### Separability analysis using Partial Least Square

Next, to inspect overall distributions of the data, PLS analysis has been performed (Figure 3). Two classes have been separated well on two principal component axes, showing the suitability of the data for this biomarker finding study. Further, these results show that urine samples contain information that can discriminate breast cancer from normal, presenting the possibility to diagnose breast cancer by acquiring and analyzing urine samples from the patients. The information contained in the urine samples is likely to be related to the causes of breast cancer.

### Feature selection analysis

Among 26,306 peaks, feature selection has been performed to extract significantly meaningful metabolite peaks between two groups of people. After many trials using different feature selection methods, 10 feature sets have finally been selected by Decision Tree (C4.5 algorithm) [16] in different datasets of 10-fold cross-validation. Among the 10 feature sets, the best feature set (Table 1A) and the second set (Table 1B) are shown here. These two sets consist of five features including one common feature (191.2261 m/z with 535.3876 retention time). The features consist of m/z valued-peaks with retention time information, and the optimal number of features in each set has been automatically selected by the C4.5 algorithm, which has pruning functions to avoid over-fitting. For comparison, a feature set (Table 1C) is selected by the univariate feature selection method (Chi-square), and the ranks in the feature list from the univariate feature selection are provided for the three feature sets.

### Construction of classification models

Using selected feature sets, classification models have been constructed through various classification algorithms in our analysis procedure with 10-fold cross-validation. In both of the two feature sets, overall accuracies were more than 90 percent. In the first set, performance, including both sensitivity and specificity, was more than 94 percent in all of the classifiers except the linear SVM. (Table 2 and 3) Finally, we have developed reliable potential diagnosis models for breast cancer based on urine samples (Table 1 and Figure 4). In addition, diagnosis rules to help in clinical decision making for breast cancer have been proposed from the models of the Decision Tree (Figure 4 and  5), and these rules can be useful to clinical applications if the proposed potential biomarkers are confirmed by further experiments. For performance comparison with univariate classification, three univariate classifiers have been constructed by each feature of the feature set from the univariate feature selection using Decision Tree with only one feature. (Table 4) These results showed that multivariate classifications outperform univariate methods by about 6.6~12.7 percent. In addition, as a semi-multivariate classification, the three features selected by the univariate feature selection have been applied to multivariate classification methods to match the number of features used in the multivariate classifications. However, in all the classification algorithms, multivariate classification methods were comparable to or also outperformed the semi-multivariate approach.

**Table 4:** Contingency table for the feature set from the univariate method

	Actual	Output		Accuracy	Sensitivity	Specificity
					
		Cancer	Normal			
**M191**						
**Univariate classification**	Cancer	46	4	87.37%	92.00%	82.00%
	Normal	9	41			
**M401**						
**Univariate classification**	Cancer	38	12	82.46%	76.00%	88.00%
	Normal	6	44			
**M311**						
**Univariate classification**	Cancer	40	10	83.12%	80.00%	86.00%
	Normal	7	43			

**M191+M401+M311** (Univariate feature selection + Multivariate classification)						
**Decision Tree** (Confidence=0.25, Pruning=true)				85.01%	86.00%	84.00%
**Random Forest** (Tree=500, Feature=6)				90.00%	90.00%	90.00%
**SVM (Gaussian)** (Cost=55, Gamma=0.33, # of SVs=17)				92.27%	96.00%	88.00%

Classification results for the feature set from the univariate method (Table 1C)					

Contingency table showing number of cases classified for each of the diagnostic classes for the feature set from the univariate method (Table 1C).

### Confirmation of multivariate classification’s power

Last, to confirm the multivariate classification’s power, a t-test, which is a univariate method, has been applied to the identified feature sets. A metabolite peak of 191.2261 m/z with 535.3876 retention time shows a p-value of 2.866e-06 in the t-test, and it seems that this peak (M191) can be identified by both the univariate and multivariate methods (Figure 6A). However, a metabolite peak of 65.21586 m/z with 687.9798 retention time (M65) shows a p-value of 0.7528, and it seems that this peak cannot be identified by only the univariate method, given the high p-value. In this case, if the multivariate method is applied, then M65 can be identified, because the multivariate method considers more than two features at the same time, although the rank of M65 in the feature list from the univariate feature selection is very low, whose rank is 4138 among 26306 (Table 1A). That is, the multivariate algorithm can find the case that breast cancer and normal samples are classified if two conditions should be satisfied simultaneously; in this experiment, the intensity of M65 is more than 117.6 and the intensity of M191 is less than 168.7. It seems that this discriminative power of multivariate methods is highly appropriate for biological systems in which more than dozens of factors are able to affect single disease. (in an algorithmic view, since M191 is a higher node than M65 in the decision tree of our constructed classification model, by a split of M191 node, all the instances are rearranged and divided into two groups so as to make the lower uncertainty of instance sets at the lower nodes than M191. Then, in M65 node, classification rules are searched using only one half of the instances). As a result, since both conditions are applied together, the criterion becomes strict and the p-value of M65 is dramatically decreased from 0.7528 to 5.478e-08 (Figure 6B). A metabolite peak of 345.2603 m/z with 1483.899 retention time (M345) has also been decreased from a p-value of 0.4299 to 0.00299 (Figure 6C).

## Discussion

To apply the proposed classification models and potential biomarkers to real clinical situations, the following analysis procedures are required:

First, additional validations must be performed using independent datasets that may have different characteristics from the data used in this study so as to guarantee the generality of the proposed models; there have been a few studies [20-22] performing validations using independent datasets. After strict validations, the metabolites corresponding to the identified peaks, which are the potential markers, must be identified. In addition, biological interpretation is required to understand why the proposed metabolites are significantly different in patients with breast cancer. This work can be accomplished by functional analysis of the metabolic pathways for the metabolites or enzymes related to them.

However, although further work remains to be done for actual application in clinical settings, this study proposes several possibilities for classification and biomarker discovery research using the urine metabolome. The first possibility is that breast cancer can be recognized by analyzing urine metabolome samples. This becomes more obvious with the results of partial least square analysis (PLS), showing that urine samples have the information that can discriminate between normal and breast cancer groups. Second, this study shows the possibility that reliable diagnosis models and potential markers, whose performance were all better than 94 percent, can be constructed from GC-MS urine metabolome datasets. After experimental validation, the proposed marker candidates will likely lead to clinically applicable assays for earlier diagnosis of breast cancer. Furthermore, this study shows the possibility that multivariate methods can discover ‘hidden features’ that univariate methods cannot easily find. It seems that this capability is very important with complex, noisy data, such as urine metabolome data that may be affected by heterogeneity in cancer progression, individual differences of genetic makeup, or the averaging of multiple characteristic signals into undistinguishable signals.

## Conclusions

Diagnostic assays based on urine samples have several major advantages, including non-invasiveness, inexpensiveness, and ease of performance, that will likely lead to impacts in clinical settings including the earlier detection of malignancies. As the end products generated by an organism, metabolites are closely linked to phenotypes and can be diagnostic clues regarding abnormalities. In this study, we have proposed analysis procedures using multivariate classification to more precisely analyze these urine metabolome data. By using multivariate classification methods, we were able to more effectively analyze urine metabolome datasets for which univariate analysis is not powerful enough due to the data’s complexity. We have found in our experiments that this multivariate approach can identify features that are not recognizable by univariate methods. In conclusion, we have proposed classification models and five potential urinary biomarkers for breast cancer diagnosis. Our findings will be helpful in real clinical settings if additional validations and experimental confirmations are performed.

## Competing interests

The authors declare that they have no competing interests.

## Authors' contributions

YK designed metabolome data analysis procedures, implemented them, analyzed classification results, and wrote the manuscript. IK co-designed the analysis procedures and advised on statistical methods. BJ and BC produced metabolome profiles and analyzed them. DL conceived this research and critically reviewed the whole data analysis works and the manuscript.
